# Good’s Syndrome: Time to Move on From Reviewing the Past

**DOI:** 10.3389/fimmu.2021.815710

**Published:** 2022-01-12

**Authors:** Aunonna Kabir, Reza Alizadehfar, Christos M. Tsoukas

**Affiliations:** ^1^ Department of Experimental Medicine, McGill University, Montreal, QC, Canada; ^2^ Department of Medicine, Divisions of Allergy and Clinical Immunology, and Pediatrics, McGill University, Montreal, QC, Canada; ^3^ Department of Medicine, Division of Allergy and Clinical Immunology, McGill University, Montreal, QC, Canada; ^4^ Division of Experimental Medicine, Research Institute of the McGill University Health Centre, McGill University, Montreal, QC, Canada

**Keywords:** Good’s syndrome, CVID, immune deficiency, hypogammaglobulinemia, thymoma

## Abstract

For seven decades, the pathophysiology of Good’s syndrome (GS) has remained a mystery, with few attempts to solve it. Initially described as an association between hypogammaglobulinemia and thymoma, controversy exists whether this is a unique disease, or a subgroup of Common Variable Immune Deficiency (CVID). Recently, some distinguishing aspects of both syndromes have come to light reflecting fundamental differences in their underlying pathophysiology. GS and CVID differ in demographic features and immune phenotype. GS is found almost exclusively in adults and is characterized by a significantly reduced or absence of peripheral B cells. In CVID, which also occurs in children, most patients have normal or slightly reduced peripheral B cells, with a distinguishing feature of low memory B cells. Similarly, differences in T cell dysregulation and manifestations of hematologic cytopenias may further distinguish GS from CVID. Knowledge of the clinical phenotype of this rare adult immune deficiency stems from individual case reports, retrospective, and cross-sectional data on a few cohorts with a limited number of well characterized patients. The understanding of pathophysiology in GS is hampered by the incomplete and inconsistent reporting of clinical and laboratory data, with a limited knowledge of its natural history. In this mini review, we discuss current state of the art data and identify research gaps. In order to resolve controversies and fill in knowledge gaps, we propose a coordinated paradigm shift from incidence reporting to robust investigative studies, addressing mechanisms of disease. We hope this novel approach sets a clear direction to solve the current controversies.

## Introduction

Good’s syndrome (GS) was initially defined as a rare association of thymoma, invasive bacterial infections and hypogammaglobulinemia, diagnosed in 40 to 60-year-old adults (1). It is one of the most unique, yet under-investigated immunodeficiencies. The initial definition of GS created a mindset that it is a subset of Common Variable Immunodeficiency (CVID) with thymoma. Subsequent evidence of T cell deficiency, autoimmune features, and myelodysplastic manifestations, suggest a more complex clinical picture. As a result, modifications to the original diagnostic criteria have been proposed to exclude non-immunodeficient overlap syndromes (2). This proposed paradigm shift stems from the lack of an established underlying etiology. Since first described by Dr. Robert Good in 1954, there has been a paucity of literature on the subject with a lack of clinical awareness, resulting in an inadequate characterization of this disease. A turning point in our understanding came following the discovery of B cells in 1960. It then became apparent that this syndrome included reduced or absent mature B cells (3). In the latest review of GS, four cohort studies reported a total of 44% of individuals who had complete absence of peripheral B cells, while 50% had low levels (4). Thus, it is appropriate that reduced or absent B cells be accepted as an additional diagnostic criterion for GS (5, 6). Furthermore, the focus on B cell lymphopenia may lead to understanding the underlying etiology.

The discovery of other thymoma-related disorders of immunity led to a blurring of the classic definition of GS. Thymoma associated disorders, including autoimmune manifestations, giant cell myocarditis, myasthenia gravis, absolute lymphocytosis, and also isolated T cell immunodeficiency of unknown pathogenesis, have created a diagnostic dilema by inappropriately defining some of these patients as having atypical GS (6–9). In this review, we summarize existing knowledge, identify the controversies and “unknowns” with possible avenues of investigations on its immunopathology. We specifically focus on the importance of clearly defining GS as an immune deficiency and stress the importance of B cell lymphopenia.

## Controversies Regarding Underlying Immune Pathology:

### Is GS a Subset of CVID?

Defects in B cell differentiation and antibody production comprise the largest group of inherited disorders of immunity (10). From a B cell perspective, GS fits between two sub-categories of primary antibody deficiencies (11, 12). The first is characterized by absent or significantly reduced numbers of B cells and all serum immunoglobulins, such as X-linked agammaglobulinemia, λ5 deficiency, BLNK deficiency, PIK3R1 deficiency, and Igα/β deficiency. The second subcategory, the CVID phenotype, is characterized by a history of recurrent bacterial sino-pulmonary infections, low or normal numbers of peripheral B cells with a moderate to severe reduction in at least 2 immunoglobulin isotypes and poor responses to vaccines. In contrast to patients with CVID, only <5% of patients with GS have normal levels of B cells (4, 13). GS is traditionally grouped with antibody deficiencies, where 100% GS patients have low serum IgG levels and 86% have low IgA and 92.6% low IgM (4). While CVID patients may have defects in peripheral B cell survival, differentiation, and antibody production, GS patients are different from CVID, and are similar to those with agammaglobulinemia in that they lack B cells, suggesting a defect or interference in lymphopoiesis during early B cell development (See **Table 1**).

**Table 1:** Key features of GS with contrasts to XLA and CVID.

	CVID	XLA	GS
Prevalence	1 per 25,000-1 per 50,000	1 per 1,400,000	1 per 700,000
	(14)	(15)	(16)
**Diagnostic Criteria**			
Thymoma	−	−	+
Hypogammaglobulinemia	+	+	+
Presence of peripheral B cells	Normal to moderate for 90% of patients (13)	Absent for 100% of patients (17)	Significantly reduced or absent for 99% of patients (16)
**Age**			
Proportion diagnosed in childhood	20%	100%	<1%
	(18)	(15)	(19)
Age range at presentation	20-40yrs	0-2yrs	40-60yrs
	(18)	(20)	(21)
**Infections**			
Invasive bacterial	+++	+++	+++
Opportunistic	+	−	++
Associated clinical conditions	ITP, AIHA, lymphadenopathy, splenomegaly (22)		PRCA, myasthenia gravis, lichen planus (16)
Frequency of autoimmune complications	~20–30%		>50% of cases
	(22)		(4)
Genetic cause identified	Identified in 10% of cases (TACI, BAFF-R, CD40, CTLA) (14)	BTK (23)	Unknown

(-/+): absence/ presence of characterstic; (++/+++): increasing frequency.

A recent study of bone marrow samples from GS patients, found that the block in B cell differentiation was at a different stage than in agammaglobulinemia patients with defined monogenic causes (XLA, μ-chain-, CD79 or BLNK deficiency) (24). While most B cell progenitors (BCPs) in the agammaglobulinemia patients were arrested at the pre-B-1 and pre-B-II stage, due to defective pre-BCR signaling, BCPs of GS arrested at the earlier Pro-B cell stage. GS patients also had a severe reduction in total BCPs compared to healthy controls and agammaglobulinemia patients. It is unlikely that a germline defect in B cell differentiation is the underlying cause because of the late onset, and reports of monozygotic twins discordant for GS (25, 26).

In addition to recurrent bacterial infections, GS patients experience an increased frequency of opportunistic infections (OIs) (mucocutaneous candidiasis, Pneumocystis jirovecii) and reactivation of latent viruses (cytomegalovirus, varicella zoster, human polyoma virus 2, herpes simplex virus) (27–30). The presence of OIs is diagnostic of significant T cell deficiency and thus, defines the syndrome as a combined immune deficiency. OIs are not a hallmark of antibody deficiencies such as XLA and CVID, however, a small subset of patients with CVID do report such infections (31). It is yet unclear if defects in cellular immunity are a feature of all GS patients or similar to CVID, they only occur in a subgroup. T cell dysregulation has been extensively studied in subsets of CVID with a wide and heterogenous set of abnormalities. Despite a higher proportion of GS patients with cellular immunodeficiency, in-depth investigations on T cell function have not been carried out to the same extent as in CVID (16).

### Is It an Inborn Error of Immunity or a Secondary Antibody Deficiency?

Monogenic defects occur in some patients with CVID, involving pathways of B cell development and differentiation, and maintenance of germinal centers (11, 32). No disease-causing variants have been associated with GS, with only three independent genetic studies being conducted so far. Out of eight patients reported, two were found to have a mutation in TACI and one individual has been reported to carry two missense mutations in BAFF-R (33–35). BAFF-R and TACI, members of the Tumor Necrosis Factor Receptor (TNFR) superfamily, are involved with B cell maturation and homeostasis, and variants of these genes have been associated with CVID. Although animal models indicate loss of function mutations in TACI which result in peripheral B cell deficiency, human single nucleotide variants are not associated with peripheral B lymphocyte changes (36). Presence of mutated variants do not confirm that these genes are in fact disease causing or even susceptibility increasing.

Furthermore, there are arguments to suggest that unlike XLA or other agammaglobulinemia disorders, GS may not be driven by a mono or polygenic cause. Most cases of CVID and GS are adult-onset. The age of CVID diagnosis has a bimodal presentation: ~20% being diagnosed during early childhood and the other set, usually between ages 20-40, often with a prodrome of recurrent infections years before their diagnosis (18). GS patients, almost exclusively, report a healthy childhood and adolescence, with the diagnosis of both thymoma and hypogammaglobulinemia, occurring between 40-60 years of age. In contrast to these adult antibody deficiencies, most cases of congenital agammaglobulinemia are diagnosed in early infancy, after 6 to 9 months, when most of the maternal antibodies have been lost (See **Table 1**) (17). The very late onset of GS, in patients who also have absent peripheral B cells and arrested progenitor B cells, highlights the importance of age as a risk factor. However, studies have not determined the roles of incomplete penetrance or mosaicism. Understanding the mechanism of disease in GS may also shed light on the role of age-dependent epigenetics in B cell lymphopoiesis.

GS is postulated to be a secondary immune deficiency (i.e. thymic tumor induced hypogammaglobulinemia) (37). The diagnosis of thymoma is usually an incidental finding or part of the diagnostic investigations for suspected myasthenia gravis, or recurrent pneumonia and bronchitis (38). In 90% of GS cases, the thymomas reported are benign and localized. One systematic review reported the spindle cell as the most common variant (WHO classification A), while others reported > 50% of cases are mixed thymomas (WHO classification AB) (21, 29). In either case, no association was found between thymoma type and type of opportunistic infections or secondary autoimmune complications. The diagnosis of thymoma can precede, occur concurrently, or follow the diagnosis of the hypogammaglobulinemia, at relatively equal frequencies (21, 29, 39).

In one large retrospective survey, the median age of GS diagnosis was 58 (51-62 years), and a median interval of four years between diagnosis of thymoma and that of hypogammaglobulinemia, irrespective of the sequence of presentation (29). Thymectomy does not reverse the immune or hematologic abnormalities in these patients (27). The timing and relative non-impact on the hypogammaglobulinemia can lead to the impression that the thymoma itself does not drive the B cell depletion in GS but is simply another clinical manifestation of the unknown immunopathology.

### Autoimmune Complications vs. Bone Marrow Dysplasia

Although both GS and CVID have hematologic manifestations, the underlying mechanisms differ. In GS, Anemia is seen in 50 to 86% of patients. Pure Red Cell Aplasia (PRCA) is the most common cause, along with aplastic, hemolytic, and pernicious anemia and myelodysplastic syndromes (4). These manifestations are indicative of bone marrow failure. In contrast, the most common autoimmune conditions in CVID, are immune thrombocytopenic purpura (ITP) and hemolytic anemia (AIHA), both are antibody mediated (40). Bone marrow dysfunction may be responsible for other hematologic defects associated with GS including lymphocytopenia, CD4 lymphopenia, neutropenia and eosinopenia (21). Lymphadenopathy and splenomegaly, seen frequently in patients with CVID, are rare amongst GS patients. The contrasting features of these two predominantly antibody deficiencies are shown in **Table 1** and may provide more insight on the intersection of genetics, autoimmunity, and inborn errors of immunity (41, 42).

Given that no genetic defect in B cell differentiation has been demonstrated in GS, there may be intrinsic and/or extrinsic factors driving the B lymphopenia. An oligoclonal expansion of a subset of CD8 T cells with a vβ8 T cell receptor (TCR) in the bone marrow of patients with a thymoma and B lymphopenia, has been reported. However, this expanded subset was not seen in the same patients’ peripheral blood lymphocyte population (43). Direct sequencing revealed a conserved CDR3 motif in the vβ8 TCR (SF/LGXGXNXXQ/LH/Y) suggesting that this could be an antigen-specific response to either an unknown pathogen or an autoimmune targeting of B cell progenitors. Several studies have shown that the thymic tumor microenvironment can cause aberrant maturation of T cell precursors and alter the T cell subset composition in the blood, but most studies are limited to myasthenia gravis. Additional hypotheses suggest the role of autoantibodies and limitin, an interferon-like cytokine produced by bone marrow stromal cells, in suppressing or skewing the differentiation and growth of B cell precursors (39).

A few small studies have reported that GS peripheral blood lymphocytes can suppress both pre-B cell differentiation and peripheral B cell differentiation to plasma cells, and subsequent immunoglobulin synthesis. Since GS patients have almost no peripheral B cells nor pre-B cells at diagnosis, their T cells or lymphocytes were co-cultured with either bone marrow cells or peripheral B cells of allogenic healthy controls. The lack of HLA matching between donors could imply that the suppressive effect was due to an allogenic response to non self MHC (25, 44–46). Of interest, in one study, the suppressive nature of lymphocytes on B cell differentiation and function, was still observed when the patient’s cells were co-cultured with those of their monozygotic HLA identical twin (25). More work needs to be done to ascertain if CD8 T cells are in-fact the drivers of the B cell lymphopenia of GS and if it is mediated by contact-based cytotoxicity or functional suppression through secretion of a soluble factor. The development of an immune response targeting self could be multi-factorial. In addition to thymic skewing of T cell subsets, an environmental “hit” could also cause an expansion of suppressive clonal lymphocytes. Several viruses and bacteria have been postulated to induce auto-immunity through molecular mimicry, epitope spreading, bystander activation and cross-reactive antibody production (47, 48). Identification of a common pathogenic agent in patients would be radical, as this would reclassify GS as an induced autoimmune disorder leading to a secondary immune deficiency and can create a shift in treatment paradigm.

## Discussion: Research Gaps & Moving Forward

In a 2003 detailed review on GS, extensive recommendations were made on investigations required to fill the gaps in knowledge (39). Almost two decades later, these gaps remain strikingly evident (see **Table 2**). The existing literature on GS, consists mainly of individual or small series of case studies. These have been focused on hospitalized patients with admissions for severe infection, short-term clinical treatment, and outcomes. To date, only two cohort studies have been conducted with only one being prospective in nature (16, 29). This, of course, reflects the very low prevalence of 1.5 cases per 1,000,000 individuals in a population per year (16). It is encouraging that patients are being recruited through primary immunodeficiency registries. However, longitudinal studies, as those conducted for CVID, are required to delineate the natural history of the GS. Moving forward with limited patient numbers, concerted multicenter efforts should include consensus laboratory and clinical investigations. A dedicated database and biobank should improve power and quality of genetic analysis and *in-vitro* assays.

**Table 2:** Existing knowledge gaps regarding Good’s syndrome and proposed solutions.

Knowledge Gaps regarding GS	Proposed solutions
**Incomplete definition of GS**	- Reaching consensus on the diagnostic criteria - Incorporation of B cell lymphopenia in the criteria - Validation and use of other biomarkers (e.g.CD247) to rule out other differential diagnosis with other thymoma associated phenotypes (6)
**Lack of published natural history of disease**	- Multi-centre longitudinal studies - Case reports detailing clinical events at multiple time points
**Low power genetic screens and *in-vivo* assays**	- Increase awareness and patient recruitment at a multinational level - Open access of real time results - Establishment of a dedicated GS database - Establishment of a blood and tissue biobank
**Lack of uniform and detailed immune characterization**	- Consensus on immunophenotyping - Longitudinal bio-marker sampling, in good health and when symptomatic - Report laboratory ranges for healthy and other immune deficiency controls - Promote the use of functional immune assessments (cytokines, lymphocyte proliferation)
**Unvalidated hypotheses regarding etiology or mechanism of disease**	- Additional bioinformatics investigations - whole exome sequencing, RNA seq, HLA typing - Screens for autoantibodies and soluble factors that interfere with B cell lymphopoiesis (e.g., limitin) - Additional laboratory investigations-culturing of thymus, bone marrow or peripheral PBMCs

The current evidence on genetics can neither confirm nor rule out that there is a monogenic cause underlying GS. It is useful to screen diagnosed GS patients for all known immune deficiency associated genes and make the results available in open access, even if no variant of significance is found. This will help narrow down potential candidate alleles that possibly occur in GS patients. Given the age of onset, the defect may be at a translational or epigenetic level. It is therefore important that whole exome sequencing, protein expression quantification and CGH microarray (looking for micro-duplications and micro-deletions) be considered. A particular tissue of interest for study is the thymus at removal. A multiomic approach should be taken, using targeted panels or whole exome sequencing, looking for somatic mutations, as well as RNA sequencing, of thymoma cells. GS thymoma cells should be compared to the appropriate controls; healthy thymic tissue or other thymoma associated diseases such as myasthenia gravis. This may provide insight on the variability in histological type of the thymomas reported in case studies (Type A vs Type AB). Similar approaches can also be taken with bone marrow samples. The purported role of CD8 T cells provides rationale for HLA typing, which has been seen to be skewed in other diseases such as CVID and myasthenia gravis (36, 49).

Adequate GS immune characterization is lacking, and if present, is difficult to reproduce. Less than half of case reports provided immunophenotyping and if available was not in a uniform fashion (21). Some reported absolute cell numbers, others reported percentages (39). Additionally, many evaluations were on a single occasion in the disease course or at an age not stated. Nor can we assume consistency of data for all individuals reported. Most case studies were of hospitalized individuals with significant infections, possibly skewing the laboratory findings. Often results were provided without context (such as normal ranges for healthy age matched controls) or comparison with appropriate disease controls (such as individuals with CVID). Only a single study compared individuals with CVID and GS prospectively, but chose to focus on clinical infections, and only included a single immune investigation at an unspecified time-point (16). There is a need for an increased awareness of this syndrome. Once diagnosed, patients should be followed regularly with standardized clinical evaluations. Given the importance of myelodysplasia, trends in neutrophils, platelets, and red blood cells over time should be monitored on follow-up and captured in a common database. Early detection documenting the onset of lymphopenia, absence of B cells and neutropenia would improve our understanding of the progressive nature, immune deficiency, and dysregulation in this syndrome.

There is a need for robust in-depth extended immune phenotyping, including vaccine challenge for humoral and cellular responses. *In-vivo* assessment using anergy screens, have been rarely reported. Beyond *in-vitro* mitogenic lymphocyte stimulation with lectins such as PHA, it is important to assess responses to microbial recall antigens, as well as post vaccination to neoantigens (SARS Cov-2). As *in-vitro* stimulation alone may not predict the susceptibility of select patients to opportunistic infections, measuring thymic output and diversity of the CD4 and CD8 TCR repertoire may also offer predictive value given the para-thymic nature of this syndrome. The multiple hypotheses arguing the autoimmune mediated repression of the proliferation of pro-B lymphocytes must also be tested. A first step in this direction would be screening for autoantibodies or other suppressive factors such as limitin in patients with thymoma (see **Table 2**).

Although informative, case studies of a cross sectional nature and subsequent reviews of the literature, have shed little light on the etiology and the underlying immunopathology. Since the initial reporting of GS, improvements in laboratory technology and bioinformatics, have been crucial in solving many medical mysteries. With the increase and almost instantaneous global sharing of scientific knowledge, we recommend sharing of databases and biobanks, given that these are quintessential assets when dealing with limited patient numbers. We foresee that if these tools and cooperative agreements are optimally deployed, knowledge gaps in GS can be closed, accelerating the understanding of the pathophysiology of this rare disease.

## Author Contributions

CT conceived and supervised the mini review. AK reviewed the literature and drafted all sections of the article. CT and RA revised and edited the draft. All authors read and approved the final article.

## Funding

This work was supported through the generosity of the Anna-Maria Solinas Laroche Allergy and Clinical Immunology Research fund and the Montreal General Hospital Foundation.

## Conflict of Interest

The authors declare that the research was conducted in the absence of any commercial or financial relationships that could be construed as a potential conflict of interest.

## Publisher’s Note

All claims expressed in this article are solely those of the authors and do not necessarily represent those of their affiliated organizations, or those of the publisher, the editors and the reviewers. Any product that may be evaluated in this article, or claim that may be made by its manufacturer, is not guaranteed or endorsed by the publisher.
